# Three Cases of Gouty Tophus in the Foot Treated by Resection

**DOI:** 10.7759/cureus.37144

**Published:** 2023-04-04

**Authors:** Taro Shiozaki, Tadashi Kimura, Mitsuru Saito, Makoto Kubota

**Affiliations:** 1 Department of Orthopaedic Surgery, Jikei University School of Medicine, Tokyo, JPN; 2 Department of Orthopaedic Surgery, Seirei Hamamatsu General Hospital, Hamamatsu, JPN

**Keywords:** hyperuricemia, resection, surgery, foot, gouty tophus

## Abstract

We encountered three cases of gouty tophus in the foot that required resection. All patients were male and aged 44-68 years at the time of surgery. The lesions were located on the great toe, second toe, and lateral malleolus and were causing ulceration and destruction of the joints. One patient had normal uric acid levels, and another patient had hyperuricemia but no history of attacks and no significant inflammatory symptoms around the gouty tophus, which was attributed to the physical containment of uric acid crystals by the gouty tophus. Given that the crystals were adherent to the surrounding fibrous tissue and cartilage surface, we resected them as far as possible to reduce the total amount of crystals and treated the remaining crystals with uric acid-lowering therapy. There were no complications at the time of surgery. The swelling and bone destruction subsided with continued medical treatment, resulting in significant improvement in quality of life. Patients with gouty tophus should be treated aggressively with medication and monitored to prevent severe joint destruction and ulceration. Excision of the nodule should be considered in cases of exacerbation.

## Introduction

A gouty tophus is a foreign body granuloma that forms around a localized sodium urate crystal precipitated from an excess pool of uric acid against a background of chronic hyperuricemia [1]. First-line treatment is a uric acid-lowering agent. Although surgery is rarely required, it may be considered in cases with infection, skin ulceration, or functional impairment [2]. This report describes three cases of gouty tophus in the foot that were treated by resection.

## Case presentation

Case one

The patient was a 68-year-old man who had been aware of swelling around the distal interphalangeal (DIP) joint of the left second toe for five years before visiting our hospital. The joint had been painful for a month and a fistula with leachate had developed. He was referred to our hospital when his previous doctor suspected osteomyelitis of the middle phalanx. His medical history was significant only for hypertension.

**Figure 1:**
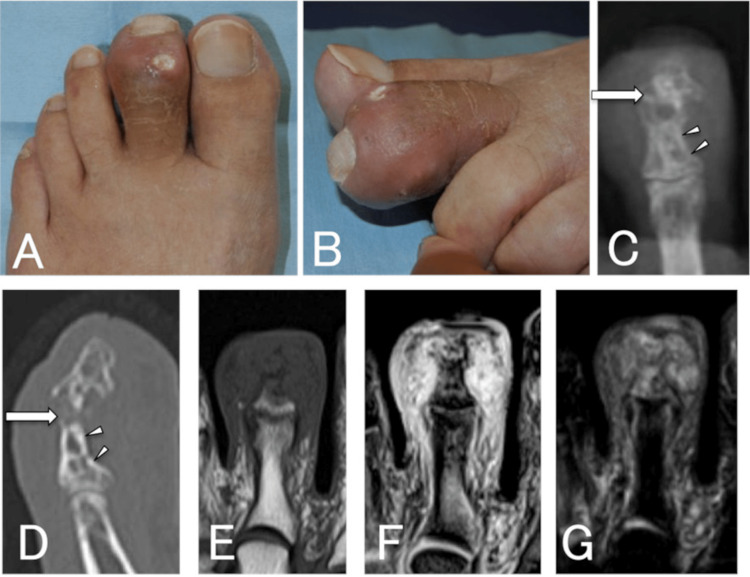
Case one: clinical and radiological findings. A, B: Photographs showing swelling of the left second toe, and powdery white contents from the ulcer exposed on the dorsal side and seen through the skin on the lateral side. C, D: A radiograph and computed tomography scan showing a fracture of the distal interphalangeal joint (arrow) and depression of the metaphysis due to the compression of the mass (arrowheads). E-G: Magnetic resonance images showing a soft tissue mass surrounding the distal and middle phalanges with a low signal on a T1-weighted image (E), a high signal on a T2-weighted image (F), and a high signal on a short tau inversion recovery image (G).

**Figure 2:**
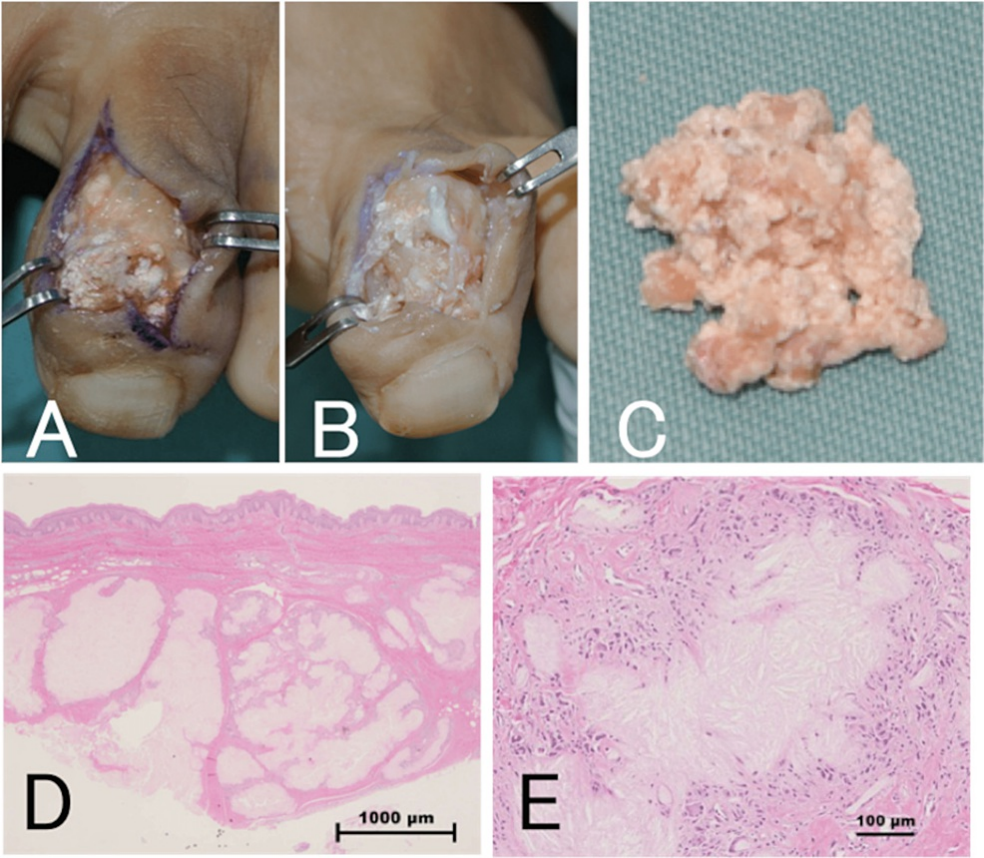
Case one: intraoperative findings and results of histopathological examination. A: Photograph showing massive accumulation of white powdery contents under the skin. B: The contents were removed as far as possible with cupped forceps. C: Excised contents. D, E: Histopathological examination revealed many pale eosinophilic nodules surrounded by heterophilic multinucleated giant cells, as well as granulation tissue with lymphocytic infiltration around the nodules.

On initial examination, there was a swelling on the left second toe and the white powdery contents of an ulcer with a diameter of about 5 mm were exposed on the dorsal side and seen through the skin on the lateral side (Figures 1A, 1B). Blood tests showed a white blood cell count of 6,800/μL, a C-reactive protein level of 8.22 mg/dL, a uric acid level of 5.1 mg/dL, and inflammatory reaction but no hyperuricemia. Plain radiographs and computed tomography (CT) images showed the destruction of the DIP joint and depression of the metaphysis, which was attributed to compression by the chronic mass (Figures 1C, 1D). Magnetic resonance imaging (MRI) revealed a soft tissue mass with a low signal on a T1-weighted image and a high signal on a T2-weighted image surrounding the distal and middle phalanges of the left second toe (Figures 1E-1G). The white content was found to be uric acid crystals on polarized light microscopy. We elected to perform surgery to prevent the progression of skin ulceration and bone erosion. A skin incision was made to extend the ulcer, and a large amount of the white powdery substance was found under the skin (Figure 2A). As there was no clear capsule formation, it was difficult to remove the contents entirely and they were resected as far as possible with cupped forceps (Figures 2B, 2C). Histopathological examination revealed multiple pale eosinophilic nodules surrounded by foreign body-type multinucleated giant cells and granulation tissue with lymphocytic infiltration, leading to a diagnosis of gouty tophus (Figure 2D, 2E).

**Figure 3:**
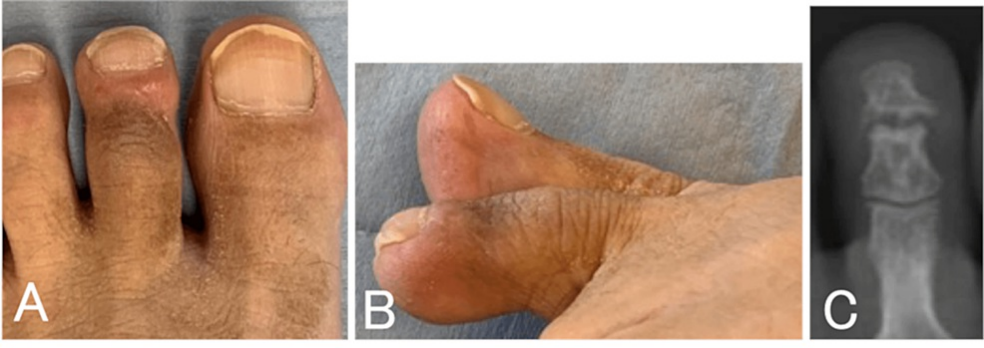
Case one: clinical and radiographic findings one year after surgery. A, B: The morphology of the affected toe is almost the same as that of the other toes. C: Radiograph showing that the bone had been remodeled.

The wound healed without inflammation. One year later, the morphology of the second toe was the same as that of the other toes (Figures 3A, 3B). Despite contracture of the DIP joint, the bone was remodeled and there was no pain (Figure 3C). Although the uric acid level was not markedly higher than before, we administered febuxostat 10 mg in the hope of absorbing the small number of uric acid crystals remaining in the affected area. The uric acid level was controlled at about 5.4 mg/dL.

Case two

**Figure 4:**
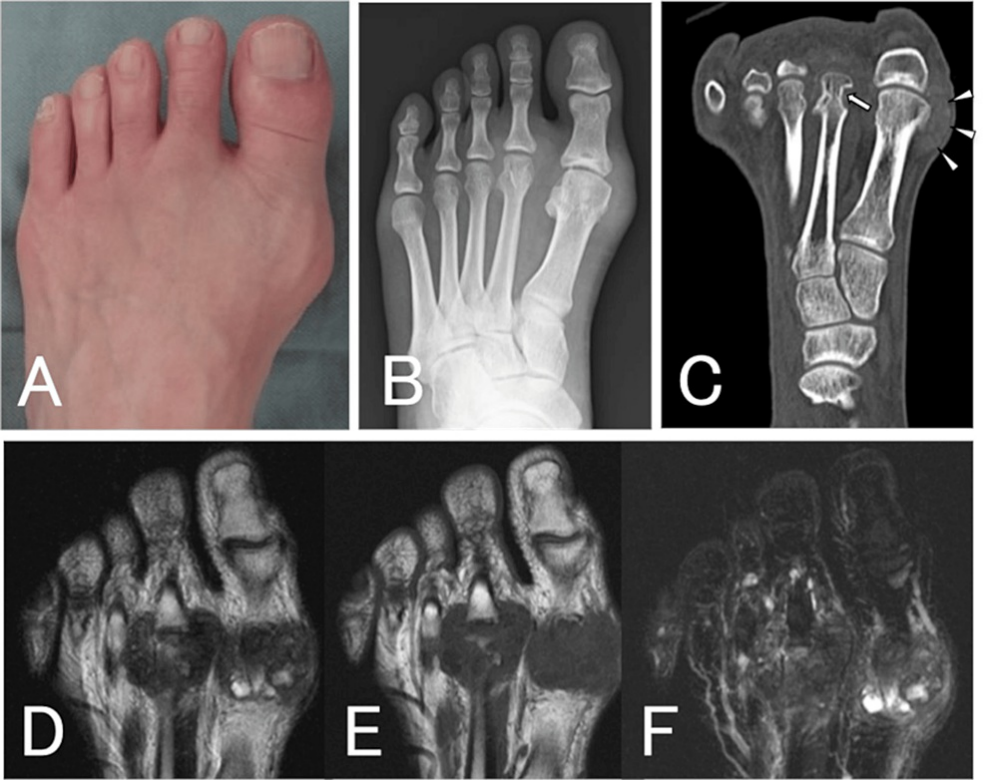
Case two: clinical and radiological findings. A: Photograph taken at the initial examination showing swelling of the metatarsophalangeal joints of the left great and second toes. B: Radiograph showed bony erosions on the heads of the left first and second metatarsals. C: Computed tomography scan showing bony erosion at the neck of the second metatarsal (arrow) and calcification in the soft tissue (arrowheads). D-F: T1-weighted (D) and short tau inversion recovery (F) magnetic resonance images showing a mixed low and high signal, and a T2-weighted image (E) showing a heterogeneous low signal, indicating a mass-like lesion.

**Figure 5:**
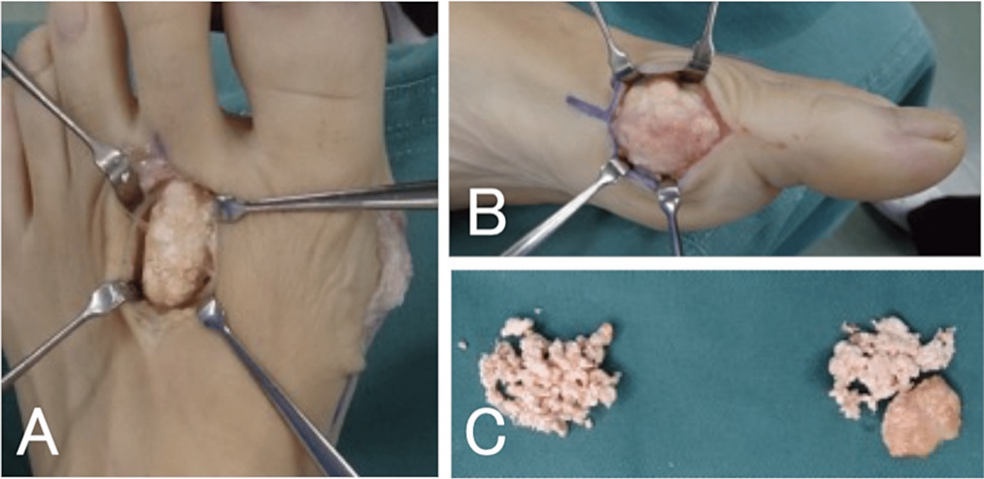
Case two: photographs showing intraoperative findings. A, B: Resection of the masses on the great and second toes with resection of as much of the surrounding scattered contents as possible. C: Excised contents.

The patient was a 44-year-old man who presented to our hospital with a painful mass in his left foot that had spontaneously appeared one year earlier. He had hyperuricemia but no history of gout and was taking febuxostat 20 mg prescribed at another hospital. Initial examination revealed pain and swelling in the metatarsophalangeal (MTP) joints of the great and second toes of the left foot (Figure 4A). Laboratory investigations revealed a high uric acid level (9.1 mg/dL) and a white blood cell count (8,500/μL) and a C-reactive protein level (0.03 mg/dL) that were within the normal range. Plain radiographs and CT images showed bony erosions in the heads of the first and second metatarsals and calcification in the soft tissues (Figures 4B, 4C). MRI showed mass-like lesions in the MTP joints of the left great and second toes with mixed low and high signal on T1-weighted and short tau inversion recovery images and heterogeneous low signal on T2-weighted images (Figures 4D-4F). The patient was suspected to have gouty tophus and underwent surgery due to severe pain and progressive bone destruction. The masses on the great and second toes were excised, and as much of the surrounding scattered contents was removed as possible (Figure 5). When the mass was incised, a milky white fluid flowed out and white crystals were found to be deposited between the fibrous tissues. The white crystals were spread throughout the joint, and the cartilage surface was uniformly covered with crystals that could not be removed. Histopathological examination did not reveal a clear crystal structure, but some needle-like shell consistent with a gouty tophus was observed. The patient went on to have an attack of gout in his left great toe two months after surgery and another at four months; however, his uric acid level remained controlled at 6.2 mg/dL with continuous oral administration of febuxostat 20 mg. One year after the surgery, the patient had no pain and good joint mobility. Two years later, he underwent carpal tunnel release surgery, and gouty tophus was found in the carpal tunnel.

Case three

The patient was a 51-year-old man who had undergone resection of gouty tophus at the left lateral malleolus at the age of 20 years. He had renal dysfunction and was taking allopurinol 200 mg in two divided doses and tablets containing 6 g of potassium citrate and sodium citrate in three divided doses. However, he had persistent hyperuricemia, and his pain and nodules continued to wax and wane at the site of the previous surgery. Therefore, the patient visited our hospital.

**Figure 6:**
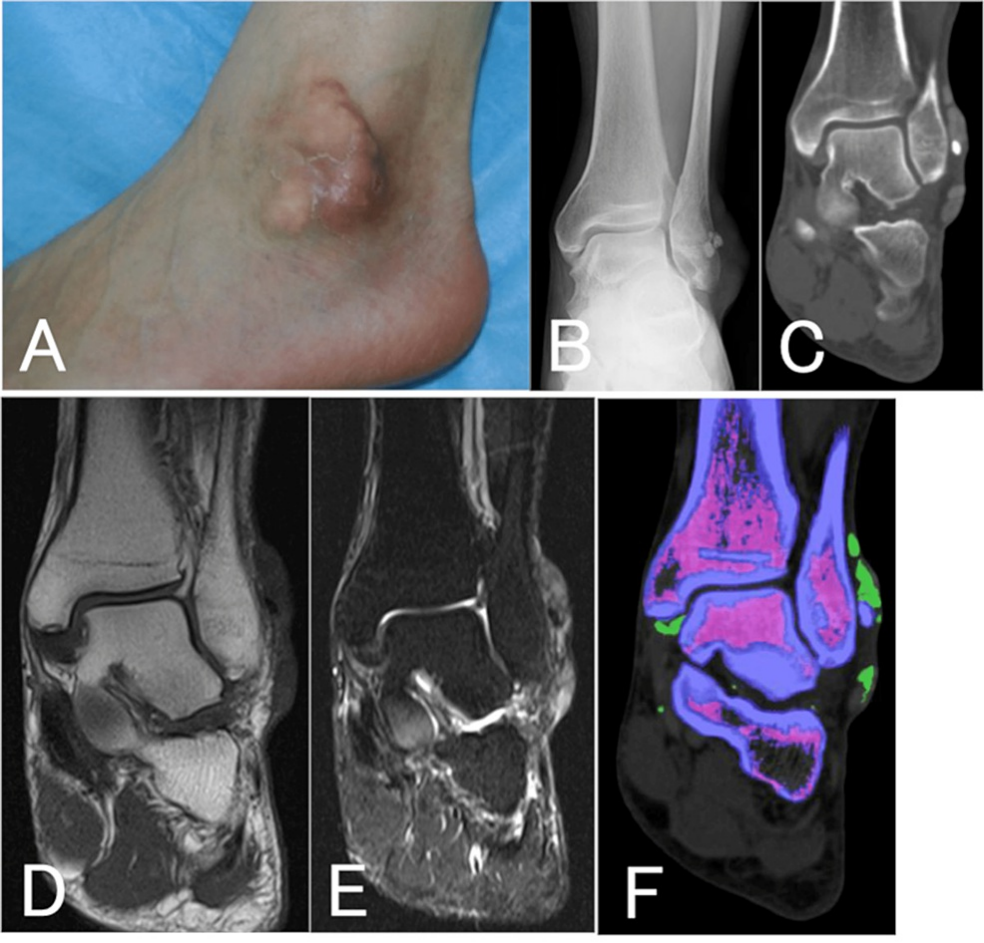
Case three: clinical and radiological findings. A: Initial examination revealed a mass in the left lateral malleolus. B, C: Radiographic and computed tomography images showing obvious calcification in the lateral malleolus with mass lesions above and below. D, E: T1-weighted (D) and T2-weighted (E) magnetic resonance images showing a mass-like lesion with a heterogeneous signal in the lateral malleolus. F: Dual-energy computed tomography scan with the gout nodule depicted in green.

**Figure 7:**
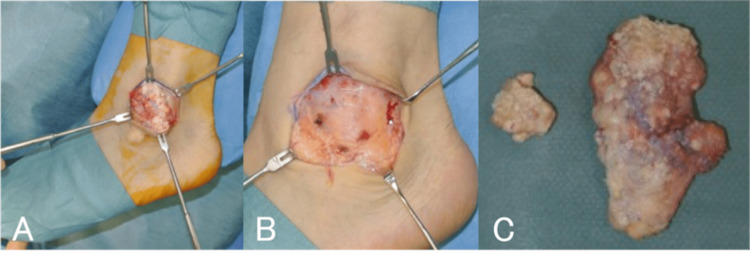
Case three: photographs showing intraoperative findings. A, B: A white, chalk-like, elastic-to-hard mass with some internal calcification was excised, including a portion of the skin. C: Excised contents.

A mass was noted in the left lateral malleolar region of the ankle joint and slight erosion of the skin (Figure 6A). Blood tests showed a white blood cell count of 9,700/μL, a C-reactive protein level of 2.14 mg/dL, and a uric acid level of 8.9 mg/dL, indicating mild inflammation and hyperuricemia. Plain radiographs and CT images showed clear calcification in the outer periphery and mass lesions above and below the calcified image (Figures 6B, 6C). MRI showed a mass-like lesion in the superficial layer of the external capsule with heterogeneous low signal on T1-weighted and T2-weighted images (Figures 6D, 6E). Dual-energy CT revealed uric acid crystals in the same area as the green-colored mass, which was distinct from the clear calcified area (Figure 6F). The patient was scanned with a SOMATOM Drive CT system (Siemens Healthineers, Forchheim, Germany) in DE mode. In view of the patient’s severe pain, we excised the mass (Figure 7). Based on the presence of crystals and the histopathological findings, the diagnosis was gouty tophus. Two years later, the patient had no pain, and the uric acid level remained at the upper limit of normal (6.9 mg/dL) even on febuxostat 40 mg.

## Discussion

The incidence of gout ranges from 9.4% to 27.1% [3,4], and gouty tophus is reported to be present in 13% of patients with gout [5]. Gouty tophus often occurs in the olecranon bursa, Achilles tendon, and phalangeal joints. As the disease progresses, white crystals accumulate and become visible under the skin, sometimes breaking through the skin and causing ulceration. Plain radiographs and CT images show bone erosion, joint destruction, and calcification in soft tissues. MRI typically shows a low-to-moderate signal on T1-weighted images and a more variable, moderate-to-high signal on T2-weighted images, which may be difficult to distinguish from malignancy. Recently, dual-energy CT, which uses X-rays with two different levels of energy, has been reported to be useful for discriminating between uric acid crystals and bone and calcified lesions [6,7]. In our third case, it was difficult to detect the gout nodule by MRI, and the diagnosis was made by dual-energy CT.

Histopathological images of a gouty tophus show a crystal mass of monosodium urate monohydrate surrounded by histiocytes, foreign body-type multinucleated giant cells and lymphocytes, and fibrosis around the mass [8]. Monosodium urate monohydrate is water-soluble and dissolves when normal specimen preparation methods are used, and in some cases, only needle-like shells can be seen on histopathology. Therefore, a histopathological diagnosis of gout nodules requires confirmation in alcohol-fixed specimens or the finding of needle-like crystals on polarized light microscopy [9].

Gouty tophi are more likely to occur with prolonged hyperuricemia and are reportedly common in patients with a serum urate level >8.5 mg/dL for more than 10 years [10]. However, case one had gouty tophus even though the uric acid levels were normal, and case two had no history of gout attacks, despite a high uric acid level and gouty tophus. Moreover, tophi can be clinically silent for a long time with no symptoms of active inflammation, indicating that the tophus may represent a physical containment that limits monosodium urate monohydrate crystal-induced inflammation to the site of deposition of crystals [1]. Recent studies have found that neutrophil extracellular traps (NETs) are involved in the formation of gouty tophus. NETs are one of the immune mechanisms associated with neutrophils and have recently been shown to be involved in a variety of diseases. In attacks of gout, NETs are removed when the crystals disappear and the inflammation subsides. However, in patients with persistent hyperuricemia, uric acid crystals remain and NETs continue to be formed by neutrophils, resulting in inadequate removal of NETs [11]. Furthermore, both pro-inflammatory cytokines from uric acid crystals and anti-inflammatory cytokines from NETs are expressed in gout nodules, and it is believed that gouty tophi enlarge without producing symptoms [12].

Gouty tophus is generally treated by uric acid-lowering agents. Continuous lowering of the serum uric acid level gradually decreases the number of urate crystals in the gouty tophus until the nodule reduces in size or disappears. Furthermore, it has been reported that the gout nodule decreases in size more rapidly when the uric acid level is controlled at a low level [2], and the recommendation for severe nodules is to aim for a serum uric acid level of ≤5 mg/dL [13]. Surgery is considered when the patient is resistant to drug therapy and has a functional disability, such as infection, ulceration, or breakdown of the skin, and cannot wear shoes [14]. In addition, osteoclasts are activated in the area where the gouty tophus is in contact with bone [15]; without treatment, bone destruction may progress, so preventive surgery should be considered. However, surgery for tophaceous gout is associated with a relatively high rate of complications. Inappropriate debridement of tophaceous lesions can result in delayed wound healing or overlying skin necrosis [16]. If the defect wound is large after gout tophus resection, continuous negative pressure therapy or skin flaps should be considered.

In our cases, the crystals were adherent to the surrounding fibrous tissue and cartilage surface, making complete curettage impossible. Therefore, we decided to reduce the total amount of crystals and hoped that the remaining crystals would be absorbed by the use of uric acid-lowering therapy and resected them as far as possible. There were no complications at the time of surgery, and the swelling and bone destruction subsided with continued medical treatment, resulting in a significant improvement in the quality of life for the patients. In case two, the first gout attack occurred two months after surgery. In this case, we believe that the crystals, which had been isolated in the nodule, may have become exposed and triggered an inflammatory reaction. Joint fixation with curettage has been reported to be effective when more than 50% of the cartilage in the first MTP joint has been lost [17]. In our patient, the destruction of the MTP joint of the toe, which is important for weight bearing, had not progressed, so curettage alone was sufficient. We believe that patients with gouty tophus should receive pharmacological therapy and be monitored to ensure that they do not develop severe joint destruction that would require joint fusion surgery and that nodule resection should be actively considered if bone or joint destruction progresses.

## Conclusions

We encountered three cases of gouty tophus that required resection. Maintaining low uric acid levels with medication is effective. Surgery should be considered as an option to prevent joint destruction and ulceration when medication is not effective and the patient is symptomatic.
